# Gallium: Assessing the Long-Term Future Extraction, Supply, Recycling, and Price of Using WORLD7, in Relation to Future Technology Visions in the European Union

**DOI:** 10.1007/s41247-025-00125-7

**Published:** 2025-07-08

**Authors:** Harald Ulrik Sverdrup, Hördur Valdimar Haraldsson

**Affiliations:** System Dynamics, Gameschool, Inland Norway University, Holsetgaten 31, 2315 Hamar, Norway

**Keywords:** WORLD7, Gallium, Sustainability, Scarcity, Peak behavior, Technology metals

## Abstract

The gallium resources were assessed and used as input to long-term simulations using the WORLD7 model. The content of gallium in different mother ores has been estimated to be about 14.7 million tons of gallium. Much of this is not accessible because of low extraction yields, about 610,000 tons gallium appear to be extractable (4%) with present practices. The gallium content in all source metal refining residuals is about 51,000 ton/yr, but only a production of 1,374 ton/yr appears as the maximum with present technology and conditions. The actual gallium production was about 450 ton/yr in 2023. The gallium price is very sensitive to increases in demand, and production is not very likely to be able to rapidly increase. The simulations show that soft gallium scarcity sets in after 2028 and physical scarcity will occur about 2060. Better gallium extraction and recycling yields may push the scarcity date forward to 2100. 60% of the gallium demand for photovoltaic technology can be satisfied in the long term. To improve the situation and prevent scarcity, extractive access, gallium extraction yields, and recycling yields must be significantly improved to better than 50%. At present, the overall yields are 7–15%. Increasing extraction yields and recycling yields can reduce the shortage. The long-term sustainable extraction is under Business-as-Usual about 300 tons gallium per year, about 67% of the present production. This poses a major challenge to future plans for an energy transition, where under Business-as-usual (BAU), such a transition will remain hypothetical. The four EEA imaginaries, Ecotopia, The Great Decoupling, Unity in Adversity, and Technocracy for the Common Good, offer different policy pathways for managing future gallium scarcity through varying degrees of technological advancement, resource conservation, and avoidance strategy.

## Introduction

Gallium is an important element for different photovoltaic technologies, semiconductors used in electronics and LED lighting technologies (Almosni et al. 2018; Bleiwas 2010). The envisioned European energy transition toward renewable energy sources implies a large amount of photovoltaic electricity production, as well as more energy-efficient technologies helping to reduce energy use. At the same time, new electronics and semiconductors depend on a larger scale on different types of gallium and on gallium in combinations with other technology metals. All of that depends on having a sufficient supply of metals like gallium for these technologies. Gallium is a secondary product of zinc, copper, lead, and bauxite mining (Kelly et al., 2014, Leveque and Helgorsky 1977, Lu et al., 2017). Other sources are being explored.

In line with the long-term sustainability vision of the European Union, the European Green Deal stresses the importance of secure access to critical minerals that are essential for the functioning of the society in the future. To this end, the European Environment Agency (EEA) has developed four alternative imaginaries that depict different visions of a sustainable Europe by 2050 (SE2050), each with different policy approaches to resource use and management (Sverdrup et al. 2024a, b). These are shaped as assumption scenarios that will need further substantiation and elaboration of details:

1. **Ecotopia:** This scenario prioritizes nature and sustainability, with a marked reduction in consumption and technological reliance. It is assumed that the public sector retracts, civil society, and local communities play a collaborative role in driving decision-making and welfare systems.
2. **The Great Decoupling:** In this scenario, it is assumed that technological innovations and social advancements have managed to decouple GDP growth from environmental degradation. Green growth is fuelled by competitive markets, with the bioeconomy at the forefront. Government interventions guide incentives, while EU cooperation remains pragmatic and focused.
3. **Unity in Adversity:** In this scenario, Europe has come together under a unified constitution to respond to environmental, climate, and geopolitical challenges. Strict economic measures prioritize environmental sustainability, with significant investments made to address climate and ecological concerns.
4. **Technocracy for the Common Good:** State control is central, with digitalization used to monitor and manage social and ecological systems extensively. The EU functions as a cohesive alliance of strong governments, driving centralized economic activity, with a shift toward deglobalization and protectionist policies.

Each of these imaginaries envisions a different role for metals and minerals, including gallium, and varies in the methods of their production, usage, and recycling. Policymakers can use these visions to craft strategies that align with the sustainability objectives of each imaginary, particularly in how critical metals like gallium can be reused and recycled (Sverdrup et al. 2024a, b). However, despite the significance of these policy visions, there is limited quantitative assessment of how the assumptions embedded in these imaginaries might affect the long-term availability and sustainable extraction of critical technology metals like gallium. This study addresses this gap by applying the integrated assessment WORLD7 model to simulate the resource dynamics of gallium under varying conditions, with a specific focus on the long-term implications of these imaginaries. This leads to a research question: What are the long-term availability limits and scarcity risks for gallium under different extraction and recycling policy scenarios, including those aligned with EEA 2050 imaginaries?

### Overview on Gallium and What it is Used for

**Fig. 1:**
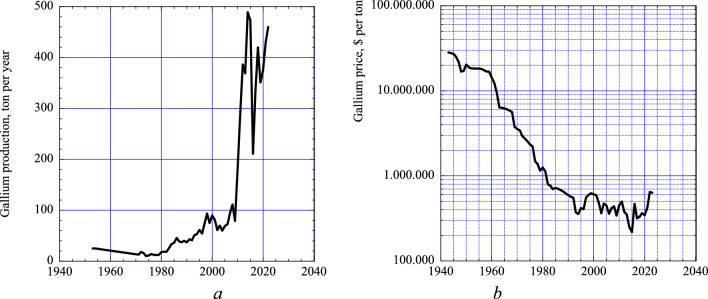
The global production of gallium (**a**). The production was historically very small (100 ton/year), but has increased strongly in the last years to about 400 ton/year. China is the dominant producer at the moment. The market price inflation-adjusted to 2020 is shown in (**b**). Data from USGS (2011, 2013, 2015, 2017, 2018, 2021, 2022, 2023) and additional data as assembled from different internet sources by the authors

Gallium is used for many new technologies (computer semiconductors, LED lighting, photovoltaic solar energy collectors, screens, advanced sensors). The supply security of gallium depend on copper, zinc, lead, and aluminum mining. Gallium are found in low concentrations in coal deposits, but a few deposits have significant amounts. Gallium is used in applications where there are no obvious good substitutes (Öhrlund 2011; Zuser and Rechberger 2011; Werner et al. 2017; Panousi et al. 2015). A eutectic alloy of gallium, tin, and indium makes an important non-toxic substitute for mercury in many of its applications. The global production in 2022 was small, only about 450 tons per year (Fig. 1a). The development of the production since 1945–2023 is shown in Fig. 1a (USGS 2019, 2021, 2022, 2023, the ds140 data). Figure 1b shows the development of the price during the same period (USGS 2019, 2021, 2022, 2023, the ds140 data). Annual production in 2022 was somewhere between 560 and 650 tons per year, all depending on the source (USGS Minerals Commodities Summary 2022; Jaskula 2023, ds140-Gallium Mineral commodity Statistics USGS website, British Geological Survey: Idoine et al. 2022). Further increase in gallium demand in the near future is expected. The Covid epidemic and the Russian invasion of Ukraine caused global disturbances in the gallium market (Jaskula 2023). China was the largest gallium producer in 2019, it largely closed production 2020–2022, and restarted production in 2023. As demonstrated in the germanium and indium studies (Sverdrup et al. 2024a, 2024b), the future availability of critical minerals is closely connected to how the pathways toward these EEA 2050 imaginaries described in Sect. 1 are manifested in policy. In particular, the scarcity of metals such as germanium, indium, and gallium poses significant challenges to advanced technologies like photovoltaics and semiconductors. The management of these minerals depends on policy frameworks that prioritize both technological and social innovations, including improved recycling, more efficient extraction, and demand-side strategies such as product lifetime extension and reuse (cf. Reuter et al. 2013a; Potting et al. 2017). The focus on innovation arises from the urgent need to close material loops and reduce primary dependency, especially in scenarios where resource intensity is expected to grow. Strategies aligned with the 10 + 3 R framework (reduce, reuse, recycle, redesign, remanufacture, refurbish, repair, recover, re-purpose, re-mine, refuse, rethink) are increasingly recognized as essential in preventing critical material shortages.

## Objectives and Scope

The goal was to address if there is a risk for gallium shortages in the future and how the proposed EU policies will address this risk or not. To do this, we need to develop an integrated dynamic model for the global market for gallium as an investigative tool. This gallium model is designed to address the following research questions:

1. What is the long-term sustainable extraction rate of gallium under business-as-usual conditions?
2. Under which conditions might soft or hard scarcity of gallium occur? (3) How can different policy scenarios—particularly those reflected in the EEA 2050 imaginaries—impact gallium availability, recycling potential, and price dynamics over time?

The gallium supply system will be explored using dynamic modeling and to asses if the supply is sustainable according to the Brundtland definition (i.e., development that “meets the needs of the present without compromising the ability of future generations to meet their own needs”; Brundtland Commission 1987). The global industrial simulations cover 1850–2200. The model is tested with respect to historical data for extraction rate and market price. The model was used to assess the long-term risk for soft or hard scarcity of the gallium in the future. Here, ‘soft scarcity’ refers to a situation where supply shortages lead to price increases and constrained availability without complete depletion, while ‘hard scarcity’ indicates a physical unavailability of the resource despite demand. The objective of the study is twofold: (1) To assemble and consolidate key data on gallium resources, reserves, production rates, and prices for use in long-term dynamic modeling; (2) To use the WORLD7 model to assess future gallium supply, identify potential scarcity conditions, and evaluate how different extraction, recycling, and policy scenarios influence long-term sustainability.

## Earlier Work

We have not found any sustainability assessment based on integrated assessment dynamic simulations for the gallium supply system and the development of the gallium supply system over time. Existing studies typically rely on static mass balances or resource inventory methods, which lack the feedback-rich analysis provided by dynamic systems models (Frenzel et al. 2016a; Lu et al. 2017; Nassar et al. 2015, Busch et al., 2014, Halada et al., 2008, Moss et al., 2011, Cucchiella et al., 2015, Zimmermann 2013). Some life cycle analysis were attemped, but in such a wat that they do not really  apply (Marwede and Reller 2014, Fthenakis et al., 2009). No considerations of the cross-linking dependencies and feedbacks in the gallium system have been published earlier or included in any model. There is no earlier process-oriented systems dynamics model for gallium. No model has earlier integrated market supply and demand modeling in the gallium flow system. Some studies have touched upon the subject using simple mass balance approaches and burn-off estimates, but these are not really valid methods for a dependent secondary extracted metal like gallium. A preliminary study was presented by Sverdrup and Ragnarsdottir (2014), Sverdrup et al. (2017) and van Allen et al., (2022). Lu et al. (2017) made a review of resources and extraction methods for gallium, stating that the gallium content in coal is about 10 million ton, that bauxite contains more than 1 million tons gallium, and that the gallium contents in zinc ores are significant. Lu et al. (2017) estimate that the gallium demand will increase from 325 ton/yr in 2017 to maybe as much as 5,000–5,500 ton/yr of gallium in 2030. This is corroborated in order of magnitude by other researchers (Candelise et al., 2011, Elshkaki and Graedel (2015), Davidsson and Höök (2015), Kim et al., (2015), and Helbig et al., (2016). If such demand growth can be met, it requires further quantitative analysis using integrated modeling approaches. Frenzel et al. (2016a, b, 2017) estimate the supply potential of gallium at 2,100 tons per year from bauxite refining, 85 tons per year from refining of sulfidic zinc ores, and potentially 590 tons per year from refining of coal combustion fly ash and slags (2,775 tons per year).

## Methods and Data Sources

### Deriving Model Assumptions from Critical Data Review

This subsection summarizes the empirical literature and data sources used to inform model parameterization, including resource sizes, extraction yields, recycling rates, and price-demand relationships. The assumptions made in the modeling are derived from previous studies (e.g., Frenzel et al. 2016a; Werner et al. 2017) and internal industrial datasets. These provide empirical boundaries for WORLD7 model scenarios, including optimistic and conservative assumptions.

The main tool employed is the system dynamics modeling method. For designing the system dynamics model, we used systems analysis. We analyzed the system using stock-and-flow charts and causal loop diagrams. The mass balance expressed differential equations resulting from the flow charts, and the causal loop diagrams were numerically solved using the STELLA®Architect modeling environment (Senge 1990; Sverdrup et al. 2022). We used causal loop diagrams for mapping out where the causalities are, to find intervention points in the system, and to propose policy interventions. The Integrated Assessment Model WORLD7 was used for this study (Sverdrup and Olafsdottir 2019). The workflow was as follows:

1. Estimate the available gallium resource that can be extracted using valid mining methods, considering the different yields and estimating the total geological deposit-to-supplied gallium approach.
2. Use the WORLD7 integrated modeling framework to estimate gallium demand from the production of consumer goods and technological infrastructures.
3. Identify the available sources of technology elements and estimate the available amounts that can be extracted from those deposits, considering extraction technologies and the cut-off caused by resource qualities and costs of extraction as related to the market price dynamics.
4. Run the WORLD7 model to assess the supply sustainability from the first use of the technology metals to about 2200.
5. Evaluate the model simulations with respect to result validity, and risk for future aspects of scarcity.
6. Evaluate how future policies that would result from the EU Imaginaries would grip into the gallium system.

The data have been stratified with respect to ore metal content and relative extraction cost (Phillips and Edwards 1976; Sverdrup and Olafsdottir 2019, 2020). For the content in coal, two approaches to estimating the content were used, both resulting in about the same amount. The gallium content in coal is low, but it remains in the ashes and gets concentrated there 20–30 times. The given yields and the resource gallium content are very approximate estimates by the authors, derived from unpublished industrial sources and in some parts from the scientific literature.

The WORLD7 integrated assessment model was used for earlier assessments supply for silver (Sverdrup et al. 2014b), copper, zinc, lead (Sverdrup et al. 2015, 2019), aluminum (Sverdrup et al. 2015), platinum group metals (Sverdrup and Ragnarsdottir 2016a, b), wolfram (Sverdrup et al. 2017c), molybdenum and rhenium (Sverdrup et al. 2018), tin (Olafsdottir and Sverdrup 2018), nickel (Olafsdottir and Sverdrup 2020), and indium (Sverdrup et al. 2023). Further publications near completion after this are studies on tellurium, selenium, yttrium, antimony, and bismuth. The source metal reserves and resources estimates for the relevant metals are based on geological estimates, the interpretation of geological data, and the allocation of extractable amounts according to ore quality, stratified with extraction costs, and as revised in our earlier studies for copper, zinc, lead, bauxite, nickel, molybdenum, and wolfram (Phillips and Edwards 1976, Olafsdottir and Sverdrup 2018, 2020; Sverdrup et al., 2021, Sverdrup and Olafsdottir 2019 (markets), Sverdrup et al. 2014a (Ag), 2015 (Al), 2017 (W), 2018 (Mo), 2019 (Ca,Zn,Pb), 2023 (In), 2024 (Ge)). The WORLD7 model take a lot inspiration to the concepts behind World3 (Meadows et al., 1972, 1974).

### Resource Estimations

#### Dependencies and Cross-Links

The different technology metals are almost all of them dependent on primary extraction of a major resource such as hydrocarbons, metals or phosphate. All the big metals and some of the minor metals but for many of the technology metals, extraction is dominated by secondary extraction. This complicated system is contained inside the WORLD7 model (Sverdrup et al. 2023, 2024a, 2024b).

#### Basis for Estimating How Much Can be Extracted from Ore Deposits

**Fig. 2:**
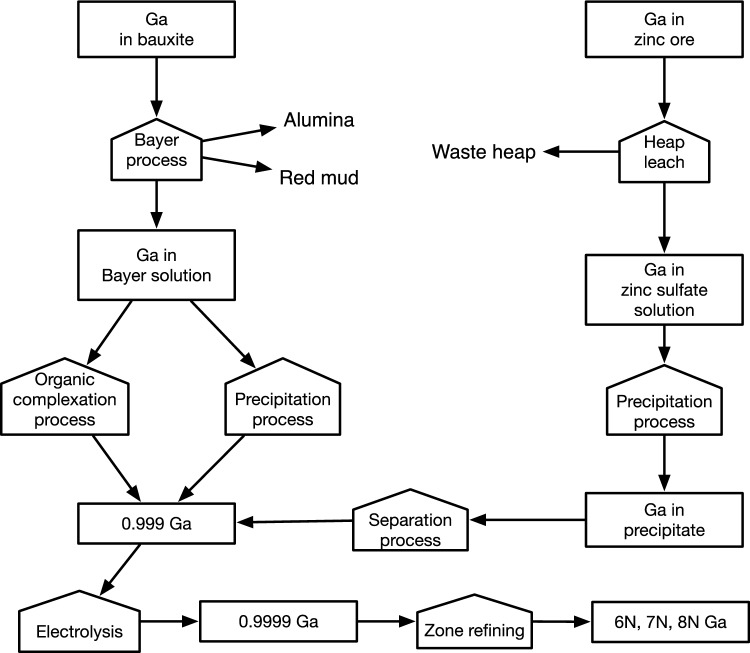
Pathway to extract gallium from bauxite and zinc ores (Raabe 2023, Rao et al., 2006). This is the main supply of gallium in 2022. There are other sources, but they are not really used at the moment (copper, lead, coal combustion ashes, see Tables 1 and 2)

Gallium is extracted mainly from Bayer Liquid from processing bauxite to alumina (about 60%) and zinc (about 40%) (Nassar et al. 2015) as illustrated in Fig. 2. A few coal mines extract some gallium from coal, and some production has been done from coal fly ash, and potentially, more is available. Gallium can be from secondary extraction during the refining process of primary metals like copper, zinc, and lead. Recycled mother metals have very little content of gallium. Gallium is only available if the ore is hydrometallurgical processed, and little technology metals come out with heap leaching methods. Only a few studies make detailed studies of the available resources of the technology metals. We have identified a few that were helpful for this study concerning: The size of the gallium resource was discussed by Frenzel et al. (2016a, b, 2017), Lu et al. (2017), Paradis (2015), Panousi et al. (2015), Wang et al. (2011) and Jaskula (2019). Statistics and data was supplied by USGS (2015, 2017, 2018, 2022). Aspects of the extraction methods and different degrees of recycling was discussed by Moskalyk (2003, 2004), Zhao et al., 2012, Zimmermann and Gössling-Reisemann 2014, and Redlinger et al. (2015). No earlier studies on gallium involve any real feedbacks from market dynamics. Other studies were read for the general picture (Phipps and Edwards 1976, Prior et al., 2012, Simandl et al., 2023, Woodhouse et al., 2013, Zhucheng et al., 2007).

Estimating the demand is particularly uncertain and was discussed by Goe and Gaustad (2014), Gibson and Hayes (2011), Dehnavi (2013), Weeks (1973), and Licht et al. (2015). The basic approach is to assume a certain percentage growth per year and let it expand exponentially. This works short term and is generally not based in any fundamental system dynamics knowledge of the gallium system. Another approach was to let it expand with GDP. The more sophisticated approaches trace the demand back to specific technology needs and derive estimates from that.

#### Extractability and Yields

One major source of difference between the available resource estimates in the literature is the differences (or lack of) assessment of actual industrial extractability. That a deposit has a certain amount of metal does not mean that it can all be extracted. The extraction pathway for a metal in general is shown in Fig. 1. We have looked at many assessments to see if they seem to converge on a similar resource base for gallium (We reviewed Werner et al. 2017; Yellishetty et al. 2017; USGS 2022; US Bureau of Mines (1941), Brown et al. 2015; Frenzel 2016; Mudd et al. 2014, 2017; Nassar et al. 2015; Sverdrup and Olafsdottir 2020, for gallium resource estimates). Yield is defined as being made up of several components: Access yield: The part of the deposits will be available for this kind of extraction.

**Fig. 3:**
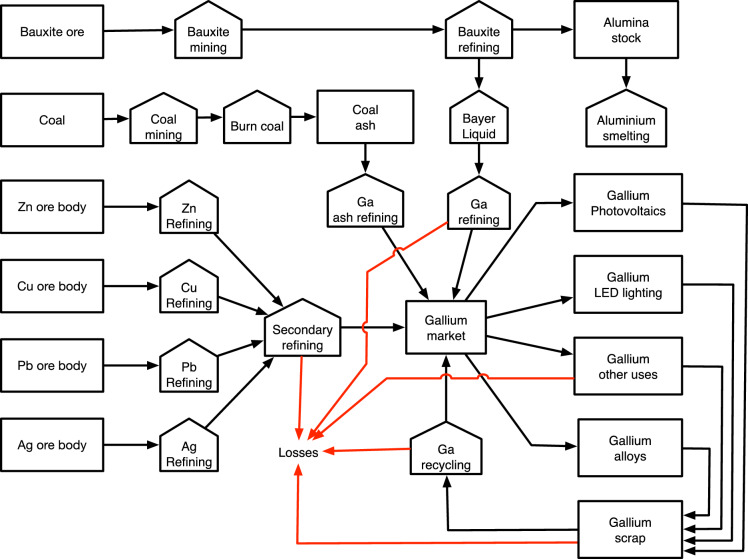
Flowchart for the submodule of the WORLD7 model dealing with gallium. The major gallium loss flows have been marked with red

Some deposits lack physical or legal access, some have a composition that prevents extraction or the extraction operation does not have the infrastructure to extract the technology metal when the operation is running Reuter et al. (2013a, b); (*Y*_A_). The substrate yield is the fraction of the potential in the source material that will be used for extraction; in many mines, the refining residuals are simply thrown away; (*Y*_S_). When the gallium is taken out, first there is an extraction where gallium is separated from the other metals present (*Y*_E_) and then it its refined into pure gallium (*Y*_R_), see Fig. 3. Some methods, such as heap leach, do not readily give such a secondary substrate that contains gallium or other metals that can be extracted. The refining yield is the fraction of the gallium recovered from the refining substrate; (*Y*_R_). Some bauxites have good gallium contents, but too much silica to be interesting for alumina production. Then gallium will not be extracted. Some coals go straight to a use where the gallium will not be extracted from the ashes. The extraction cut-off is dependent on technology, extraction costs, and the metal price at the time.

#### Resource and Extractable Amounts Estimation

The recoverable resources are estimated (Krautkrämer 1988; Sverdrup and Olafsdottir 2019, 2020):

1 $${\text{Gallium}}\;{\text{resource}} = {\text{Mother}}\;{\text{metal}}\;{\text{resource}}*X_{{{\text{Ga}}}} *Y$$

2 $${\text{Gallium}}\;{\text{production}} = {\text{Mother}}\;{\text{metal}}\;{\text{production}}*X_{{{\text{Ga}}}} *Y$$

*X*_Ga_ is the fraction gallium in the material, and *Y* is the yield (Singer 2007, Krautkrämer 1988; Sverdrup and Olafsdottir 2020; Sverdrup et al. 2023). This is defined as

3 $$Y = Y_{{\text{A}}} *Y_{{\text{E}}} *Y_{{\text{R}}}$$

The refining yield *Y*_R_ will be a function of the extractive efficiency when treating the ore shipped to the refinery. The material contained below the cut-off grade is lost with the waste (Krautkrämer 1988; Sverdrup and Olafsdottir 2020). The extraction yield *Y*_E_ is

4 $$Y_{S} = { }\frac{{\left( {{\text{Substrate grade }}{-}{\text{ extraction cutoff substrate grade}}} \right)}}{{\text{Substrate grade}}}$$

where *Y* is the overall yield, the amount of metal extracted divided by the total metal content in the original ore. The extraction cut-off is determined by the technology used for extraction, combined with several cost aspects of extraction. Gallium can be extracted from the waste liquid when bauxite is processed to alumina (Bayer process) and if the extraction plant has the necessary infrastructure for it. Some Russian and Chinese coal deposits are known to contain significant amounts of gallium. The different estimates come from different sources, not using the same background material, and they are thus not always consistent. Gallium extraction, before 1950, was very small. Yields from refining residuals are far higher than the access yields and enrichment yields, suggesting that investment in technical ability for gallium extraction appears to be missing. Possibly because on the individual process step level, each process is difficult to make profitable, even if gallium is very important on the whole-system strategic level.

This way of estimating the long-term average supply sets a final date for the resource, and after this time, it will potentially no longer be available at all. Take note that “sufficient need” is not necessarily the same as “want” or “demand”.

## Dynamic Simulation Model Description

**Fig. 4:**
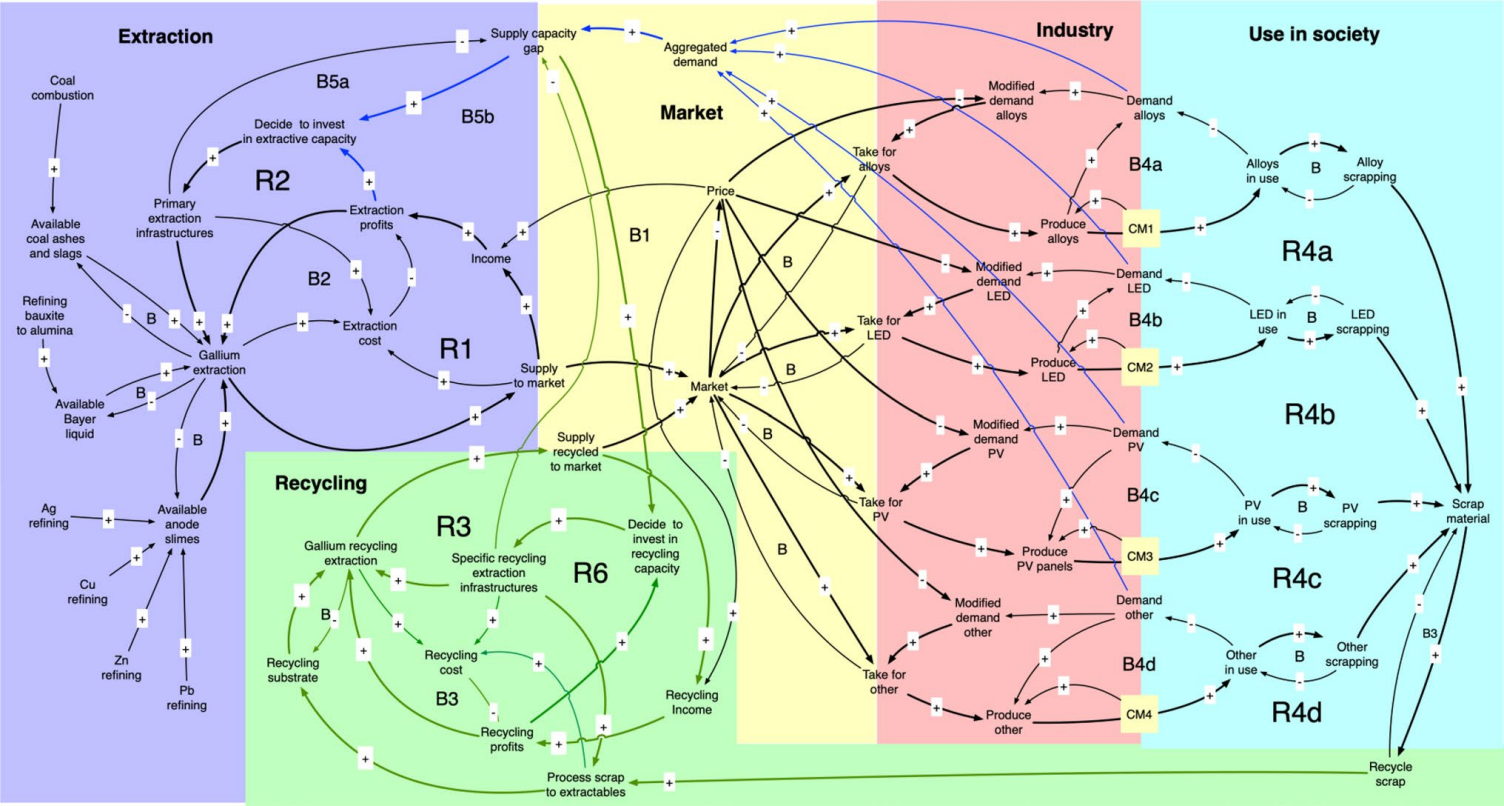
Causal loop for the gallium sub-model. R1, R2, and R3 are reinforcing loops driving the system. R1 is the extraction loop, driven by profits, R2 is the extraction-investment loop, describing how increased infrastructure increases production and profits. R3 is four different loop running over recycling to generate income and recycled gallium which is sold again to the market

**Fig. 5:**
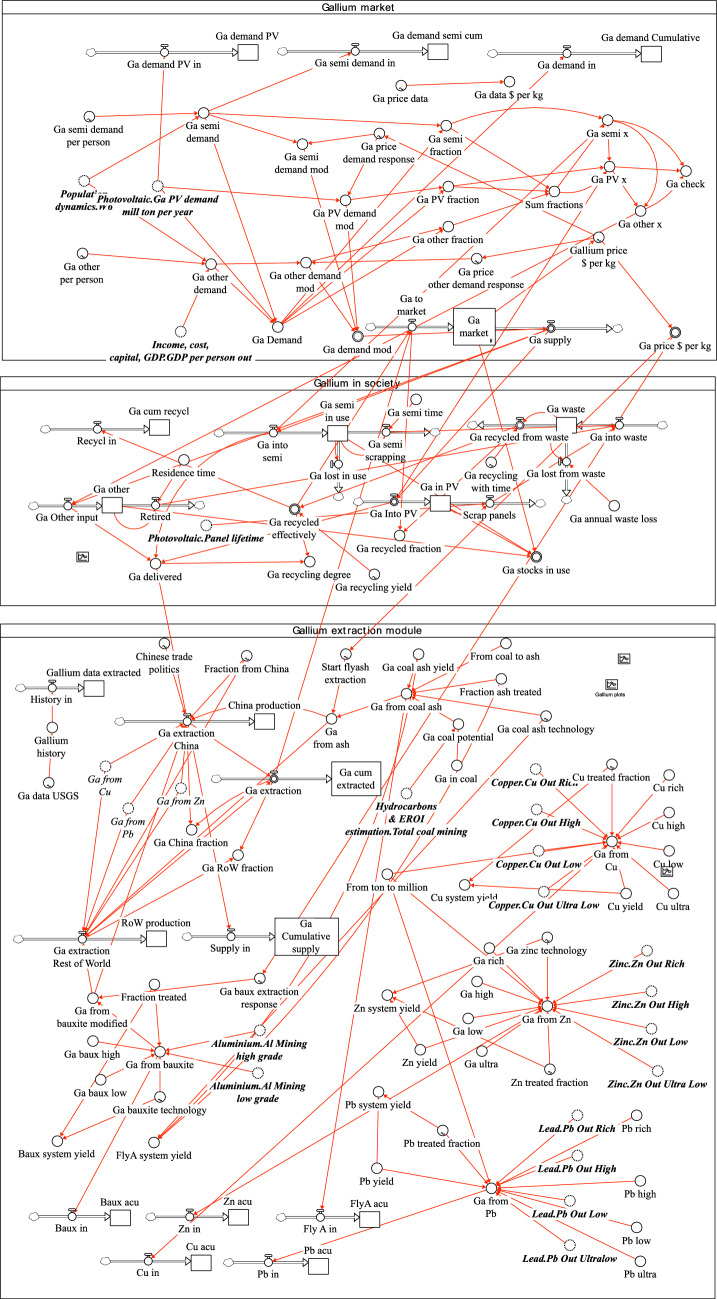
The STELLA Architect code inside WORLD7 for the gallium module

The dynamic model is best described by the system flow chart as described in Fig. 4 and the causal loop diagram (CLD) shown in Fig. 4. Figure 3 shows the flow chart for the part of the WORLD7 model dealing with gallium. The major gallium losses have been marked with red. In the model, tin was ignored as a source of gallium, as the contribution to the total supply is small. Figure 5 shows the causal loop diagram for the gallium sub-model. In certain aspects, the CLD has been simplified in order to keep it readable. Market with blue is the extractive sector, in green is the recycling sector, in yellow is the market, red is the industry using gallium in commodities, and turquoise is the use in society.

The system feedbacks for system irreversible losses of gallium have not been shown. The system driving the preparation of gallium extraction substrate from coal ash, Bayer liquid, and anode sludges from primary metal extraction has not been shown. R1, R2, R3, and R4 are reinforcing loops driving the gallium supply system. R1 is the extraction loop secondary to primary production of metals, and it is driven by profits. R2 is the extraction-investment loop, describing how increased extraction infrastructure increases extraction production and extraction profits. R3a and R3b are the loops for investing in recycling capacity. These links have been indicated in green. The investment decision is being taken based on profits and a supply capacity gap. These links have been indicated in blue. R4a–R4d are four different loops running over recycling to generate income and recycled gallium which is sold again to the market. R6 is the recycling-investment loop, describing how increased recycling infrastructure increases recycling production and recycling profits.

B are different balancing loops, slowing the system down. B1–B4 are different balancing loops, slowing the system down. B1 is the balancing effect of a large supply to the market, which may press the market price down. B2 is the balancing effect of operation costs and capital cost of invested capital in infrastructure for first extraction of gallium from different substrates. B3 is the balancing effect of operation costs and capital cost of invested capital in infrastructure connected to recycling of gallium. B4a-B4d are the balancing effect of supply and demand in the system to deliver commodities with gallium to society. B5 is the balancing effect of closing the supply capacity gap by investing in more capacity. The small unnumbered B’s are stock depletion balancing loop in the system. The system involves a supply chain from extraction substrates and recycling substrates through extraction processes to the metal market, and then to users of gallium for inclusion in commodities. The commodities flow to society and after use to scrap, where a part becomes a recycling substrate, closing the cycle.

In the WORLD7 model, take from the market is driven by the modified demand, put gallium into society where it stays until scrapped or removed by wear and losses. Demand in the model is driven by population and metal use per person, but is adjusted up or down with price; this is the modified demand. The demand is estimated from average affluence, expressed as disposable income per person and global population, using outputs of the WORLD7 model (Sverdrup et al. 2019; Sverdrup 2019). The basic driving mechanism of gallium extraction comes from profits of the extraction activity and availability of an extractable substrate used in the model. The price is determined by how much metal is available in the market in the same way as in our earlier metal models. This approach has been very successful in endogenously modeling the price (Sverdrup and Ragnarsdottir 2014; Sverdrup et al. 2017b; Sverdrup and Olafsdottir 2018, 2019). A high metal price will increase profits and promote larger supply to the market. High price leads to a reduction of demand in the model. More supply to the market will increase the amount available and lower the price.

The WORLD model was developed from 2012 and is still ongoing (Sverdrup et al. 2023, 2024a, 2024b). The WORLD7 model addresses a large number of metals, and they are all in some way linked in their extraction. All modules in WORLD7 are interconnected. The WORLD7 energy module supplies energy from fossil fuels, renewables, and nuclear power, with a market price generated by supply and demand in the model. Energy for metal extraction is taken from this module (Sverdrup et al. 2019).

Table 1 shows average estimated use of technology metals, gram/unit; indium, gallium, antimony, selenium and silver for CIGS (CuInGaSe) photocell technology, and some other technologies. gram/unit. The copper, zinc, lead, and silver production was derived in the WORLD7 model (Sverdrup et al. 2015, 2019; Sverdrup and Ragnarsdottir 2014). The aluminum module in WORLD7 was used for the contribution from bauxite mining, integrated as a submodule into the WORLD7 model (Sverdrup et al. 2015). Coal production and fly ash production rates were derived in the WORLD7 energy module. For gallium, the price has an effect on demand, but not any significant impact on the supply, as this is dependent on the source metal extraction rate. There is no strong feedback from gallium production and back to copper mining in the industry. The price has effect on demand in the model, but it does not have any significant impact on the supply, as this is dependent on the mother metal extraction rate. The mining cost depends on the energy price, other operating costs, and financial costs. The scrapping process for stock-in-use in society is driven by price (Dahmus and Gutowski 2007; Sverdrup and Olafsdottir 2019); after the metal has been scrapped, a high price will promote more recovery. Figure 5 shows the STELLA Architect code inside WORLD7 for the gallium module.

**Table 1:** The use of technology metals, gram/unit; indium, gallium, antimony, selenium, and silver for CIGS (CuInGaSe) photocell technology and other technologies. gram/unit

Item	Indium	Gallium	Antimony	Selenium	Silver
Computers	0.040	0.002	0.770	0.050	0.250
LED TV	0.003	0.005	0.710	0.015	0.450
Mobile phones	0.0003	0.0005	0.071	0.002	0.045
CuInGaSe PhotoVoltaic	0.120	0.120	0	0.120	0.080
Cars	0.300	0.05	5	0.100	0.200
Handheld tools	0.002	–	0.140	0.005	0.010

## Results

### Resource and Production Estimates

Table 2 shows the recoverable resources of source metals in million tons of metal; this was used as input data to the WORLD7 model (Sverdrup and Ragnarsdottir 2014; Sverdrup et al. 2017a, 2017b, 2017c; Sverdrup and Olafsdottir 2018, 2019). Note how the yields are very low. Of a geological presence of 15 million tons gallium, only 0.6 million tons are estimated to be obtainable for use (4%).

**Table 2:** Resource estimates for gallium

Source	Resource (mill ton)	Rich	High	Low	Ultra	Ga content (ton)	*Y*_A_	*Y*_E_	*Y*_R_	*Y*	Extractable Ga (ton)
		ppm					Yield (fraction)				
Zinc	2,676	60	30	20	10	107,000	0.5	0.2	0.9	0.090	9,633
Copper	4,020	40	20	10	5	80,400	0.5	0.2	0.9	0.090	7,236
Tin	120	30	15	7		1,800	0.4	0.2	0.7	0.140	252
Lead	3,015	50	30	20	5	60,300	0.5	0.2	0.9	0.090	5,427
Silver	2.4			50	30	120	0.6	0.2	0.95	0.110	14
*Coal*	*420,000*	*8*				*3,360,000*	*0.1*	*0.2*	*0.8*	*0.016*	*54,000*
	*500,000*		*6*			*3,000,000*	*0.1*	*0.2*	*0.8*	*0.016*	*48,000*
	*530,000*			*4*		*2,120,000*	*0.1*	*0.2*	*0.8*	*0.016*	*34,000*
	*1,450,000*	*8*	*6*	*4*		*8,480,000*				*0.016*	*136,000*
Coal ash	58,000	200	150	100	-	8,700,000	0.1	0.2	0.8	0.016	139,000
Bauxite	112,000	80	50	25	5	6,000,000	0.5	0.2	0.8	0.080	448,000
Sum						14,949,000	0.04	609,592			

There are no primary mines for gallium. All extraction is secondary. Numbers in italics for coal grades (136,000 ton) are parallel estimates to the bulk estimate on coal combustion ashes content (139,000 tons or 136,000 tons). Take note that the yields are very low for most pathways

Table 3 shows the extraction rate estimates for gallium, assuming presently applicable substrate access and extraction yields. Gallium occurs on the average as 50 ppm in bauxite (aluminum ore) and can be extracted when the bauxite is processed to alumina, the raw material used for aluminum production (Fig. 2 and see USGS 2018). The refining yield is estimated to be at best 40% (Jaskula 2013). In reality, the real industrial yields end up far below that so far. The content in zinc ores varies from 8 to 320 ppm. Our own estimate of the bauxite resource is 112,500 million tons (Sverdrup and Ragnarsdottir 2016a, b). At 50 ppm gallium content (50 g/ton), that corresponds to a geological resource of 6 million tons gallium (Table 2). But assuming that to be all present in geological deposits to be fully extractable is wrong. The different access and extraction yields must be considered, and at present, with the best available technology, about 450,000 tons of gallium would be extractable. Most of the gallium supplied in 2023 comes from Bayer liquid from processing bauxite to alumina, see Tables 3, 4 (Ronguo et al. 2016).

**Table 3:** Extraction rate estimates for gallium, assuming presently (2023) applicable substrate access and supply chain yields. There are no primary mines for gallium, everything is secondary extraction

Source	Mining mill (ton/yr)	Rich	High	Low	Ultra	Gallium content (ton/yr)	*Y*_A_	*Y*_E_	*Y*_R_	*Y*	Extractable gallium (ton/yr)
		ppm					Fraction				
Zinc	17	60	30	20	10	340	0.5	0.4	0.9	0.090	62
Copper	22	40	20	10	5	220	0.5	0.2	0.9	0.090	20
Tin	0.4	30	15	7	–	6	0.4	0.2	0.7	0.140	0.8
Lead	3	50	30	20	5	60	0.5	0.2	0.9	0.090	5.4
Silver	0.027	–	–	50	30	2	0.6	0.2	0.95	0.110	0.2
Coal	4,200	8	6	4	–	25,200	0.1	0.2	0.8	0.016	403
Fly ash	118	200	150	100	–	17,700	0.1	0.2	0.8	0.016	283
Bauxite	350	80	50	25	5	17,500	0.5	0.2	0.8	0.080	600
Sum	–	–	–	–	–	61,028	0.026				1,374

**Table 4:** Extraction rate estimates for gallium, but assuming better yields than at present (2022)

Source	Mining rate	Gallium content	*Y*_A_	*Y*_E_	*Y*_R_	*Y*	Extractable gallium	Extraction 2023
	Mill (ton/yr)	ton/yr	Assumed future yields, as fraction of total content present				ton/yr	
Zinc	17	340	0.6	0.65	0.9	0.35	119	60
Copper	22	220	0.6	0.65	0.9	0.35	77	30
Tin	0.4	6	0.6	0.65	0.9	0.35	2	–
Lead	3	60	0.6	0.65	0.9	0.35	21	3
Silver	0.027	2	0.6	0.5	0.9	0.27	1	–
Coal	4,200	25,200	0.15	0.5	0.9	0.07	1,764	20
Fly ash	118	17,700	0.15	0.5	0.9	0.07	819	50
Bauxite	150	7,500	0.5	0.5	0.9	0.23	1,679	287
Sum	–	51,028	0.09				4,482	450

Still, they are quite low. The technical extraction yield in 2022 was about 25%. Only 13% of the potential was extracted

Table 4 shows approximate extraction rate estimates for gallium, but assuming better yields than at present (2022). With gallium, the main issue is not the size of the resource, but (1) the access to the flow of substrate for extraction from mother metals, (2) dependence on the suitability of the bauxite for alumina production (It must have a low silica content), and (3) if the infrastructure for extracting gallium is present at the site where the secondary residual material is produced. Production at present is far below the potential available (Ronguo et al. 2016). The yield in the refining step is 75–80% (Hoang et al. 2015). The different yields given in the literature are not at all consistent. The yields indicated by the USGS (Foley et al. 2017) are in the range of 10% access, about 20–30% yield in the first steps of extraction and 80% yield in the final refining to pure gallium, resulting in a total yield of about 5–10%.

The observed low yields are a major challenge in the extraction of gallium, but it seems that they may be slowly improving. Low yields imply high costs for the gallium recycled and that much gallium goes to waste. It can be seen from Table 3 that the extraction potential is 51,000 ton/year of gallium, but that because of low yields under business-as-usual, the real extraction potential is 1,340 tons per year. There is a significant potential source of gallium in coal, and that could be extracted from the ash after combustion, the ash is about 5–20% of the coal, thus gallium is enriched 5–20 times in the ash as compared to the raw coal. Earlier estimates are in the same order of magnitude as we have indicated in Tables 2 and 3. The total extraction potential is about 4,500 tons per year of gallium according to Table 3. The average reserve content was adapted from unofficial information found in company websites, confidential information from consulting companies, and industrial information. These are independent sources from USGS and similar literature (USGS 2011, 2022, Idoine et al., 2022), but the estimates are of the same order of magnitude as in Table 4 (Sverdrup et al. 2017a).

Table 5 shows the present production and capacities for gallium production. The available estimates are not internally consistent and the numbers are approximate estimates made by the authors. The final column shows a very approximate estimate of the extraction potential, and this can be compared to the present production of gallium. The gallium potential without China is about 1,540 ton/year, or three times the present production. China supplies 84% of the global supply, followed by United States at 7%, and all the rest with only marginal contributions. The present global gallium technical production capacity (800 ton/yr) is far larger than the actual gallium production (450 ton/yr) in 2023, and far less than the gallium extraction potential (3,450 ton/yr).

**Table 5:** Present production and capacities for gallium production in 2023

Country	Primary gallium production 2023, ton/yr	Estimated primary gallium production capacity (ton/yr)	High purity gallium refining capacity (ton/yr)	Alumina primary production, million (ton/yr)	Zinc primary production, million (ton/yr)	Gallium approximate potential assuming 50 ppm content and 50% yield (ton/yr)
China	380	650	?	76	4.3	2,000
Australia	?	?	?	20	1.3	525
Brazil	?	?	?	11	0.2	302
India	?	?	?	7.4	0.8	410
Peru	?	?	?	0	1.6	40
USA	30	150	320	4.3	0.8	125
Bolivia	0	0	0	–	0.8	20
Mexico	0	0	0	–	0.7	18
Ukraine	4	5	–	?	0.1	
Russia	5	5	5	0.6	0.3	22
Belgium	0	10	3	–	–	
Japan	0	10	3	–	–	
Slovakia	0	10	3	–	+	
South Korea	0	5	2	–	–	
Germany	0	5	5	?	+	
Other	?	?	59	2.0	2.0	100
Sum	450	800	400	120.0	14.0	3,540

Amounts are in ton gallium per year. The final column shows an estimate of the potential? Implies no data were found

Table 6 shows a comparison of gallium resources estimates for anode sludge residuals and potential gallium production, at different yields for recycling and extraction from anode sludges, mostly from sulfide ores. Anode sludge arises when the mother metal is refined using electrolysis.

**Table 6:** Comparison of gallium resource (ton) estimates for **anode sludge** residuals and potential gallium production (ton/yr) at different yields

Item	Extraction or recycling under different scenarios for yield (*Y*) as fraction				
	*Y* = 0.04	*Y* = 0.1	*Y* = 0.2	*Y* = 0.3	*Y* = 0.4
Probable resource (ton) (Table 1)	94,000	470,000	940,000	1,410,000	1,880,000
Production (ton/yr) (Table 5)	350	875	1,750	2,625	3,500

Table 7 shows a comparison of gallium resources in coal ash and potential gallium production (ton/yr) at different yields. Present yield from anode slimes is in the range 10–15% (Table 1). Present yields for gallium extraction from coal and coal ashes are in the range 2–4% (Table 1), gallium recycling from urban scrap is at present very low, less than 10% (Data extracted from Dahmus and Gutowski 2007, Gutowski et al. 2016, Kluczka 2024 and Reuter et al. 2013a,b).

**Table 7:** Comparison of gallium resource (ton) in **coal ash** and potential gallium production (ton/yr) at different yields. The yields marked in italics are probably out of reach

Item	Extraction or recycling yields (*Y*), expressed as fraction				
	*Y* = 0.02	*Y* = 0.1	*Y = 0.2*	*Y = 0.3*	*Y = 0.4*
Probable resource (ton) (Table 5)	140,000	870,000	*1,740,000*	*2,610,000*	*3,480,000*
Production (ton/yr) (Table 2)	500	2,500	*5,000*	*7,500*	*10,000*

Table 8 shows a summary of gallium resource (ton) and potential gallium production (ton/yr) at different yields for extraction and recycling. Increasing yields in both the extraction supply chain for gallium and for the recycling recovery chain has a large effect on the gallium supply, at constant substrate use (anode slimes and coal ashes). Table 8 shows that gallium supply can be potentially increased significantly, if higher recycling yields can be achieved. If extraction and recycling yields can be raised to 30% instead of the 4% valid at present, gallium supply could increase from the present maximum potential of 1,375 ton/yr to about 9,821 ton/yr. For extraction from anode slimes, this is probably feasible, but at a significant energy cost; for extraction from coal and bauxite, a yield of more than 10% appears to be challenging (Wang et al. 2011).

**Table 8:** Summary of gallium production (ton) at different yields for extraction from all sources and including recycling with different yields

Item	Extraction or recycling yields (*Y*), expressed as fraction					
	*Y* = 0.02	*Y* = 0.04	*Y* = 0.1	*Y* = 0.2	*Y* = 0.3	*Y* = 0.4
Extraction from anode slime, ton/yr	160	350	875	1,750	2,625	3,500
Supply from coal ash, ton/yr	500	1,000	2,500	2,500	2,500	2,500
Supply from bauxite, ton/yr	150	300	750	1500	2.250	3,000
Sum	810	1,650	4,277	5,750	6,875	9,000
Recycling yields	*R* = 0.02	*R* = 0.04	*R* = 0.1	*R* = 0.2	*R* = 0.3	*R* = 0.4
Recycling, ton/yr	16	69	475	1,438	2,946	13,500
sum	826	1,719	4,752	7,188	9,821	22,500

### WORLD7 Model Simulations

**Fig. 6:**
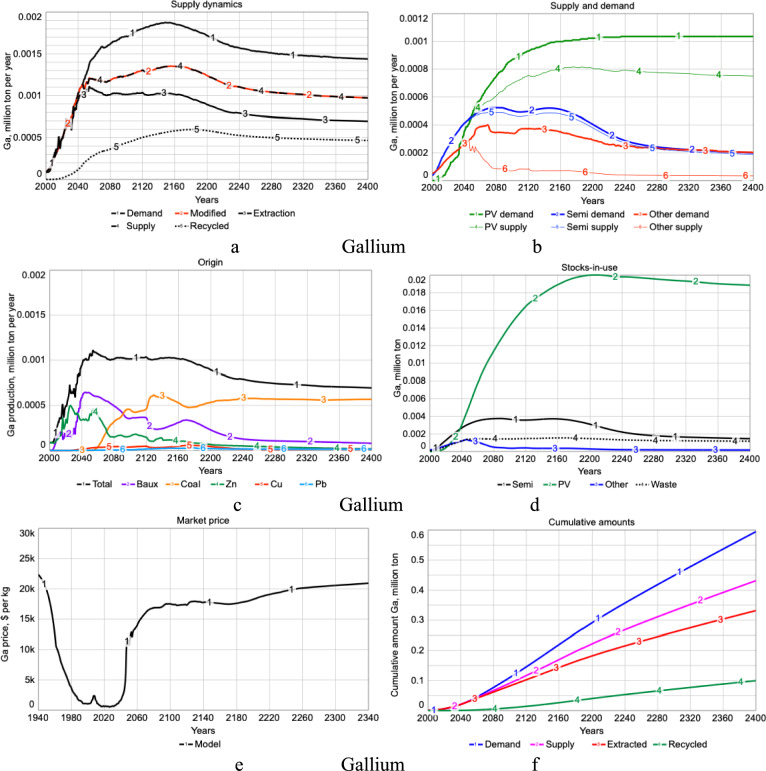
**a** The simulated production rate of gallium, together with the demand, price-modified demand, the supply, extraction, and recycling. **b** shows the comparison of demand and actual supply for photovoltaic technologies, semiconductors,and other uses. **c** shows the simulated extraction from different mother metal ores. **d** shows the stocks in use in photovoltaics, semiconductors, other use and as waste. **e** shows the simulation of the price and the amount gallium in the market. **f** shows the cumulative amounts for demand, supply, extracted and recycled gallium

Figure 6a shows the simulated production rate of gallium, together with the demand, price-modified demand, the supply, extraction and recycling. Before 2010, gallium demand was modest but that increases sharply after 2010. Figure 6c shows the extraction rate from different metal ores. The gallium supply from zinc, copper, and lead has a maximum capacity in 2055. Technological development has been assumed from 2025 to increase yields. Figure 6b shows the demand and supply into PV, semiconductors, and other uses. The production rate for gallium is shown in Fig. 6c, together with the contribution from bauxite refining to alumina, from zinc production, from copper production, from lead production and from extraction from coal ash as compared to the recorded mining history (USGS 2014, 2022). At present, gallium is mostly produced during bauxite processing in China, a preparation for aluminum production, but other sources like refining residuals from zinc and silver are becoming more important with time. Demand is driven by electronics and solar photovoltaic panels production, coming from the energy module in WORLD7.

Figure 6d shows the stocks in use in photovoltaics, semiconductors, other use, and as waste. Figure 6e shows the simulation of the price and the amount gallium in the market. After 2055, the market is nearly empty and the price shoots up. Gallium is then in hard scarcity. If extraction access and refining yields cannot be significantly improved, it will stay like that. If overall total yields are improved to better than 50%, then there will be probably no scarcity for gallium.

Figure 6f shows the cumulative amounts for demand, supply, extracted, and recycled gallium. It can be seen that demand given by Line 1 far exceeds the supply given by Line 2. The simulations suggest a long-term running average shortfall of 33% of the demand for gallium, thus only a 67% satisfaction of demand. In the long-term simulations, recycling reached only 13–15% when based on market mechanisms alone. For demand to be met in the long run, the long-term recycling flow back into the system will have to be at least 55% of the gallium supply to the market. This would require something more than just market mechanisms (e.g., gallium price) to promote recycling.

**Fig. 7:**
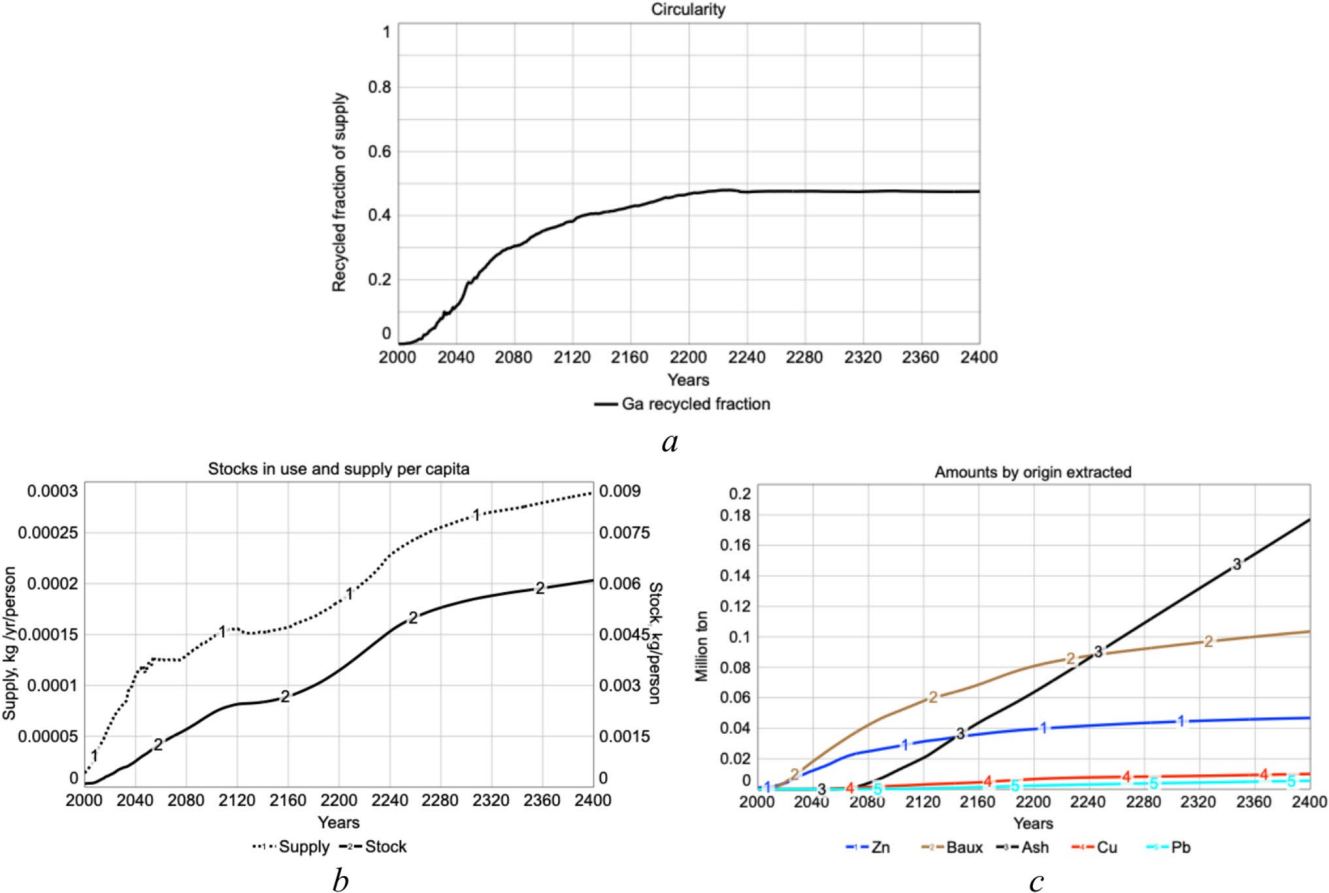
**a** Fraction of the supply as recycled gallium. **b** Gallium supply and stocks-in-use per person and year. **c** Cumulative amounts of gallium extracted from different sources over time

Figure 7a shows the fraction of the supply as recycled gallium. The recycling has been modeled as a function of price (Dahmus and Gutowski 2007; Gutowski et al. 2016). Yields for recycling are far too low and must be improved for recycling to be an important source for gallium supply. Figure 7b shows the gallium supply and stocks-in-use are predicted to increase steadily until 2400 when it peaks and later declines. Figure 7c shows the cumulative amounts of gallium extracted from different sources over time. Extraction from zinc anode slimes is dominating at the moment, but we expect that bauxite and potentially fly ash may become the dominant sources in the next decades. At present, gallium overall yields are at about 10–15%. In the simulation, dynamic recycling is depending on price.

### Testing the Model

**Fig. 8:**
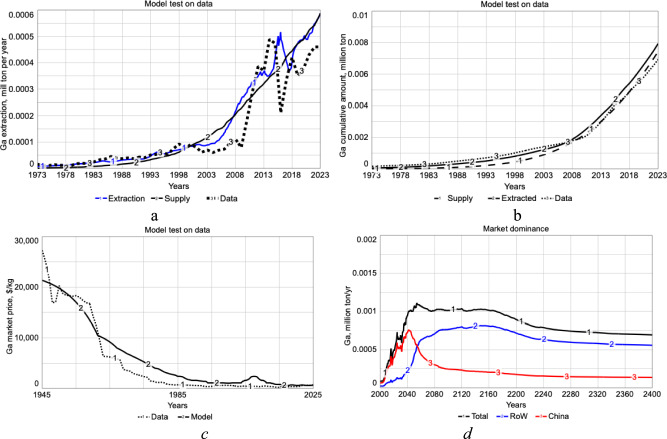
**a** shows the simulated rate of gallium extraction as compared to the observed. **b** shows the cumulative amount observed and simulated. **c** shows the simulated price as compared to the available data. **d** The flows through RoW and China and the market dominance. By 2050, the Chinese dominance over the market may have disappeared

The model has been tested against the recorded mining data derived from the USGS (2022), Foley et al. (2017) databases, as well as a continuation of earlier work (Sverdrup and Ragnarsdottr 2014; Sverdrup et al. 2019, 2023, 2024a, 2024b), as shown in Fig. 8. The general trend was to reproduce the observations, but some of the short-term variations were recreated less well. However, no systematic bias builds up over time in the simulations, as seen from Fig. 8b. The model does reproduce the observed mining rates satisfactory when the model is driven by market demand and price dynamics. The metal market price is well simulated for gallium as seen in Fig. 8c. This suggests that the model reconstructs the historical pattern reasonably well and that it is safe to use the model for exploring future scenarios.

## Discussion

### Uncertainties

The major uncertainties in the simulations come from the following aspects:

1. Uncertainties in future demand. Most of what is out there has been naive guesses by market economists based on growing future GDP. A few technical assessments of future gallium needs based on energy needs are available, but these are only partial.
2. Uncertainties in gallium content in mother ores and fly ash on a global scale
3. Uncertainties in extraction yields (substrate access, technical yields) at present and in the future

For the contents of gallium in different substrates, there is no earlier in-depth assessment. We have found numbers for gallium contents in different references, but these give approximate estimates. The situation is similar for access and extraction yields, we have some reports of gallium yields, but no assessment for the whole industry. The values found show a wide range. Thus, the yield estimates are very approximate.

The gallium reserve and resource estimates were made by the authors pieced together from the cited literature and sources. Our resource estimates are based on assumptions, but seem to align in order of magnitude with other studies. Little information is available on reserves and resources on a global level. It is known that for gallium, the content potential in bauxite is not fully used.

Many demand estimates found on the internet or in some publications are naive estimates based on a belief in constant annual growth of GDP or purely guesses about monotonous economic growth. These are normally not so useful and have been ignored. All of this is rounded off in the test on field data. The model does appear to reconstruct the past reasonably well. This indicates that the uncertainties have been kept under control and that the sum of all of it remains small.

### On Scarcity

The gallium may fast run into physical scarcity because the price has no feedback on the extraction rate of the source metals, such as on copper and zinc. For the extraction from bauxite, the economic feedback on bauxite mining rates is absent in the system. The value of the gallium per ton of bauxite is rather insignificant, and for the producer of alumina from bauxite, the extra value of the gallium obtained will be marginal. For the extraction from coal or coal ash, the drivers are strategic as much as it is an issue of profitability and operable access to an interesting substrate. At many places where coal is used, there are no conditions for extracting gallium.

Supply per person and year reflects the amount available to compensate for continuous losses and any surplus available for growth in the stock-in-use as shown in Fig. 8c. Stock-in-use per person is an indicator of the utility gained from the resource, and a decline in stock-in-use suggests a decline in service provision from that resource. Stocks-in-use is the amount providing utility. The supply is to maintain the stock, replace losses, and any surplus will cause growth; a deficit will cause a decrease in utility.

The production of gallium is limited by only a fraction of the metal refineries being technically equipped for efficient recovery of gallium from Bayer liquid or from anode slimes from Cu–Zn–Pb–Ag electrolytic refining. Light Emitting Diode (LED) lighting technology is rapidly spreading as mass products in consumer lighting devices use gallium. This demand alone could cause gallium to be physically scarce before 2060.

### On the Pathways of Gallium Through the World

**Fig. 9:**
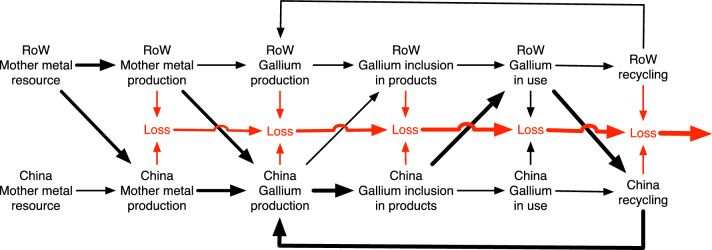
The flow chart shows the flow of gallium through the Chinese and Rest-of-the-World (RoW) systems. For long-term stability, the net primary input must amount to the growth plus the irreversible losses in the system (The red arrows). The thickness of the arrows indicates how much flows in different ways

Figure 9 shows a flow chart for the flow of gallium through the Chinese and Rest-of-the-World (RoW) systems. China is a large producer and exporter of gallium, but this occurs to a large degree from mother metal refining and mother metal refining residuals source from the Rest-of-the-World, more than Chinese domestic resources. China has had a successful strategy for securing the raw materials for making gallium, prioritizing the supply for its own manufacturing industry and then supplying the global market with those products (Han et al. 2015; Hayes-Labruto et al. 2013; Su 2009). In continuation, a lot of scrap is flowed back from the Rest-of-the-World to China for recycling, just like as illustrated in Fig. 9. If the authorities in China were to restrict the export of gallium metal that would limit gallium availability in the rest of the world. It does not really matter for global supply situation, if the gallium withheld gets incorporated into products anyway and those products would be recycled outside of China. Then, there would only be a delay. But that is not the situation at the moment. It appears that a very substantial part of the value chain has been located in China. The reasons for this are many, as the policy to do so has in the past been promoted both by Chinese and European governments and policies. For China, this puts it in a very good business position with strategic strength, as well as a political will to use strong control. Figure 6f shows the cumulative amounts of demand, supply, extraction, and recycling. It can be seen that the demand is far larger than the supply. Increasing recycling to 80% by 2030 would decrease the gap, but cannot close it. The amounts of gallium extracted 1850–2250 give an indication of what eventually will be the total available amount. Figure 9 shows the flows through RoW and China and the market dominance.

### From Problems to Industrial Opportunity

A number of problems have been discussed with respect to gallium extraction and supply to industry (and to society). For the international mining and extraction industry, these problems lead to a number of challenges, which when taken on, will lead to good business opportunities. The Rest-of-the-World has a second chance to capture gallium as it is recycled from the products, however with a delay. To do that, a strategy for capturing gallium-containing scrap and recycling it will be necessary. China does not have the world’s largest geological deposits of gallium, and thus, China only has an apparent near monopoly on gallium at the present time, because the rest of the world allows them to have it. If the gallium was produced where the deposits are and where the scrap is appearing, then the situation would be a very different one. There is some industrial capacity to do gallium extraction and recycling in Europe at present, but it would have to be significantly expanded in the near future. The EU has discussed that they should have such a strategy, many discussions are being held, but as of 2024, there is nothing real in place besides general visions and goals, and a worked-out strategy is still missing. The creation of favorable conditions for recycling businesses in Europe is not yet implemented. This would have to include proper incentives as well as protection against unfair competition and the effects of state-sponsored competition from outside countries.

Low yields in most industrial gallium transactions are a problem at present. With research into modern separation methods, these can surely be improved, leading to improved extraction yields. Such improvements often translated straight down to the operating profit of a refinery. The poor recycling rates at present are partly linked to low yields when extracting from source substrates with low content. Another challenge is one of creating a circular value chains for gallium, where the system issues of collecting and refining the scrap contain gallium. The large producing countries of alumina from raw bauxite and the major producers of zinc are listed in Table 4. Of these countries, only China and the United States have a substantial capacity for producing gallium. Every country with a large alumina production or substantial zinc production, but no primary gallium production, is potentially a business opportunity overlooked. China has at present a very dominating position in the gallium supply chain and market because of a long-term strategy and having taken their opportunities seriously. Because of the large-scale outsourcing of industrial production to China from Europe and North America, it is also one of the major users of gallium for photovoltaic technologies and LED lighting products. In the past, Chinese strategies on Rare Earth Element supply is well known, and this should be considered when the global dependence on China for gallium supply is strategically evaluated for the future.

### On future Resource Policies

**Fig. 10:**
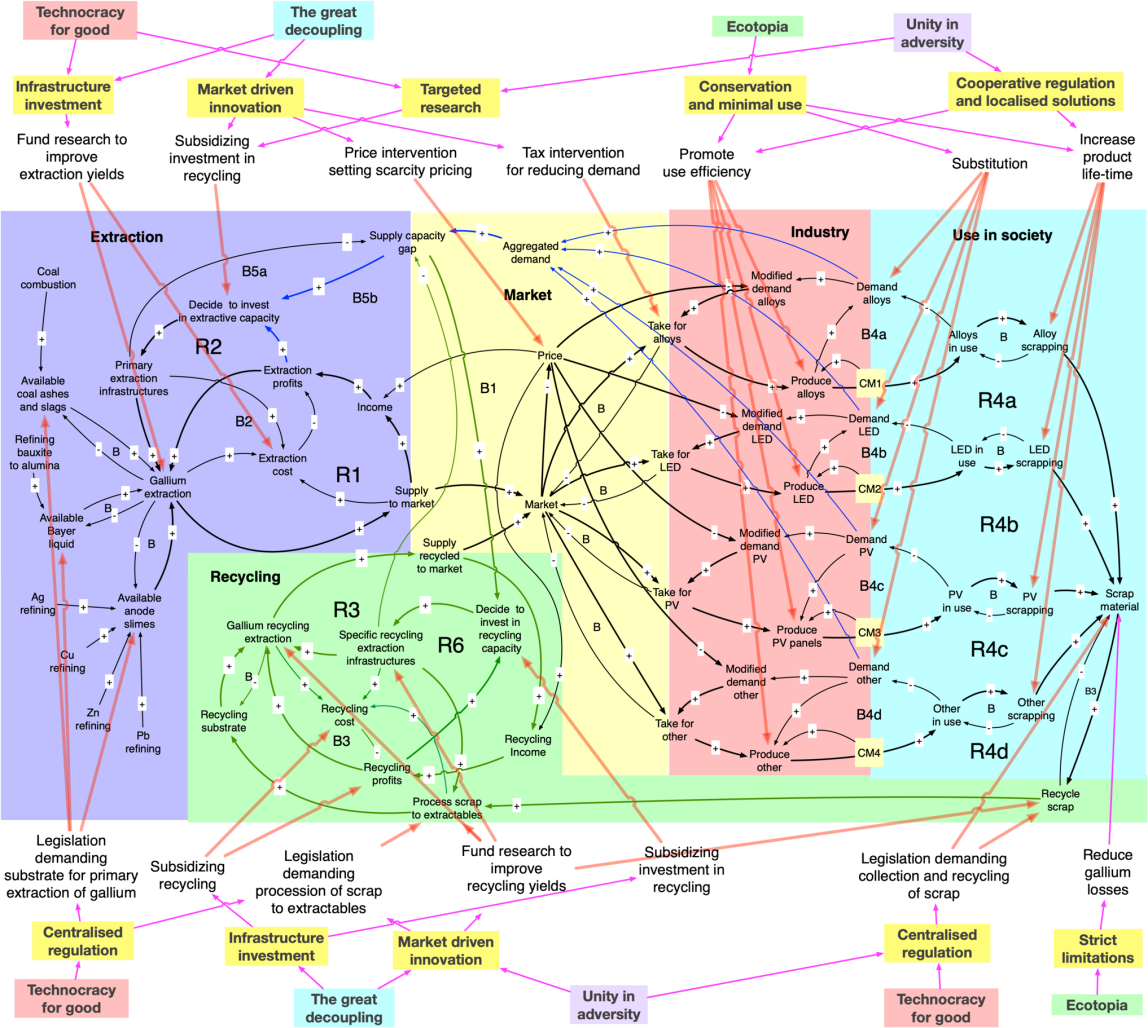
The gallium system with policy interventions including connection to the EEA imaginaries. A systemic approach is required and necessary when planning policies for complex systems. The gallium system is a very complicated system with significant delays inside where linear or short-term thinking is useless

Figures 10 shows the gallium system with examples of generic policy interventions included toward the non-predetermined future imaginary path, as well as policy intervention in realizing pathways toward the different imaginaries. A systemic approach is required and necessary when planning policies for complex systems. The gallium system is a very complicated system with significant delays inside where linear or short-term thinking does not work, especially when understanding different policy choices and their influence toward different imaginaries pathways. Planning toward sustainability without a predetermined future state (non-imaginary) results in a policy that is perceived as sustainable but without a future state. The purpose of the imaginary analysis, in this context, is to be able to frame the vision of the future and connect to the contemporary policy gap in relation to current “failed” policies. This is demonstrated in the study by the authors on indium, cadmium, and germanium (Sverdrup et al. 2023, 2024a, 2024b). It is important to consider that when the commodity is still relatively cheap, it may for that reason be unnecessarily wasted, when it would in retrospect have been relatively easy to induce better use efficiency and recycling. To wait until there is severe scarcity would be too late for optimal mitigation as unnecessary resources would already have been wasted by then.

There are ways to increase the gallium availability for society, and push the gallium supply peak further into the future by a significant amount. The following statements can be made for different policy options that may appear as possible:

1. **What does not really work that well as problem solution**
  - **More mining**: Increasing the rate of gallium extraction to satisfy demand will be great in the short-term relieving short-term problems but will not solve the root cause of the problem. More extraction from primary sources of gallium will make the gallium supply peak time appear earlier and aggravate scarcity later. Many geological surveys and mining companies are focused on this approach, mostly for short-term profits and lack of long-term impact assessment. For future generations, this is consuming the potential resources for future generations now before they are born. That is in conflict with the Brundtland and UN definition of sustainability.
  - **Secure key resource potentials anywhere in order to secure national supply exclusively**. This will foster increased risk for resource conflicts and promote the use of non-democratic methods and favor the rule of the strong to abuse the less powerful. May cause short-term local benefits but will create other problems a bit later. This solution appears to have some similarity with policies of colonial approaches.
  - **Gallium substitution** in key technologies may be a policy to consider, but with care. Substitution of gallium may appear as challenging, many of the proposed substitutes are other scarce technology metals and do not improve the overall technology metal supply challenges at all. Only when gallium is substituted with a resource in a significantly larger supply than gallium, and when this metal is not stressed by a demand in significant overshoot of the supply, will substitution make sense.
2. **What constitute parts of a solution:**
  - **Improve extraction supply chain yield**: Increasing gallium secondary extraction yield is a component of a working policy. It allows for more gallium to be taken from the same amount of extraction substrate. This shifts gallium from potential loss to potential use.
  - **Improve recycling recovery chain yield**: Increasing recycling yield is a policy component. It allows more gallium to be taken from the same amount of scrap. Increasing gallium recycling yield may have demands for how technologies (Photovoltaic technologies) using gallium are designed and that they may have to be prepared for recovery (Sverdrup et al. 2023). This shifts gallium from potential loss to potential use.
  - **Improve both extraction yield and recycling yield** Increasing recycling yield is a policy component. It allows for far more gallium to be supplied from the same amount of secondary substrate and ores, while reducing systems losses. Significant investment in research would be needed. Increasing access yield, to extract more gallium from streams that have not extraction, will convent a loss flux to an available flux, without shifting the depletion date forward. This shifts gallium from potential loss to potential use.
  - **Adaptive management of the global demand** by reducing total demand and increasing use efficiency in society (less losses, longer use time, making less gallium do the same job as more would do earlier) would contribute to the results of policy and help push the supply peak to a later date. Could possible reduce use to sustainable levels. Would need strong incentives and an active policy with some global agreements

For an effective solution, a combinations of policies would be required, by combining policy 3: Improve both extraction yield, policy 4: increasing recycling yield, with policy 5: Adaptive management of the global demand.

To further plan for sustainable management within the bounds of sustainability, the use of integrated assessment modeling will be required and WORLD7 is such a model. Further research is needed to make policy impact assessment using biophysically consistent Integrated Assessment Models, to check if proposed policy packages actually deliver the goals intended and that adverse side-effects stay under control and withing socially acceptable boundaries. Proceeding with policy proposals without doing proper and valid impact assessments is unprofessional and potentially damaging governance. Integrated models that are mass and energy consistent would be needed. Present econometric economic model that is not thermodynamically compliant will not do. Statistical interpolations will not do for the same reasons. In present policies, no consideration of future generations is taken seriously, even if it gets long speeches and beautiful works in working documents.

### Long-Term Sustainable Supply of Gallium

This section analyzes how different EEA 2050 imaginaries (Ecotopia, Decoupling, etc.) would affect gallium supply strategies. It links each policy imaginary to key model parameters: extraction yield, recycling rate, and demand-side measures. The discussion explains how the imaginaries act as qualitative scenarios informing resource governance choices. Sustainability includes both the present and future generations. Table 9 shows the estimation of critical extraction for gallium based a long-term perspective of 165 generations (5,000 years) and 330 generations (10,000 years). Under business as usual, the sustainable supply of gallium is about 100–200 tons/yr. Significantly improving access yields, mining yields, extraction yields, and recycling yields may potentially increase sustainable gallium supply to approximately 600–900 tons/yr. In the assessment, we have assumed that the “sufficient need” is the same as the “demand.” As indicated in Fig. 7, under BAU, resource extraction and technological development follow the current trends without significant changes in policy intervention. This means moderate innovation in recycling and extraction yields, but a heavy reliance on continued extraction of primary metals as a part of a sustainable strategy. As the demand for critical metals like gallium increases, BAU is at risk for resource shortages due to insufficient rate in advances in recycling, efficiency, and avoidance. Profit-driven incentives dominate and risk creating lagged and response-driven approach to long-term sustainability planning, which runs closer to resource depletion and price volatility, where market forces struggle to cope with scarcity.

**Table 9:** Estimation of critical extraction and global critical supply for gallium based a long-term perspective of 165 or 330 generations

Item	Extraction or recycling yields (*Y*), expressed as fraction				
	*Y* = 0.04	*Y* = 0.1	*Y* = 0.2	*Y* = 0.3	*Y* = 0.4
Probable resource, anode slime, ton	94,000	470,000	940,000	1,410,000	1,880,000
Probable resource coal	280,000	870,000	870,000	870,000	870,000
Probable resource bauxite	120,000	240,000	480,000	720,000	960,000
Sum available resources	494,000	1,580,000	2,290,000	3,000,000	3,710,000
Critical extraction, 330 generations	100	316	458	600	742
Critical extraction, 165 generations	200	632	916	1,200	1,484

Present yields are in the range 10%. for recycling and extraction yield, whereas yield from coal ash is less than 5% As a comparison, the supply in 2023 was 430 ton/yr

The implications toward the different imaginaries are where the imaginaries contrast sharply with BAU, where Ecotopia minimizes reliance on metals by reducing overall technological demand and avoidance/substitution approach. The Great Decoupling scenario embraces technological growth, promoting a bioeconomy-driven market approach and progress through improved recycling and extraction efficiency. In Unity in Adversity, environmental and geopolitical challenges are addressed by securing domestic supply chains and investing heavily in recycling and sustainable extraction of gallium. Emphasis is on coordinated environmental regulations and protectionist policies to safeguard gallium resources. Going step further, the Technocracy for the Common Good, Gallium, and other critical metals are tightly controlled through monitoring of extraction, recycling, and use. Government-managed resource allocation prioritizes efficiency, with centralized economic activity and protectionism securing strategic resources within the EU. Each of the imaginaries envisions a different role for critical minerals and varies in the methods of their production, usage, and recycling. Policymakers can use these imaginaries to develop strategies that align with the sustainability objectives of each imaginary, particularly in how critical metals like gallium can be reused and recycled (Sverdrup et al. 2024a, b).

### Needs for Further Research

Some of the tables shown in this study have a lot of question marks. This illustrates that there is a need for a better mapping of geological occurrence, extraction potentials, technical capabilities, and recycling yields. Some scenarios not explored here are the outcomes of different global population scenarios. If the global population does not start to significantly decline after 2100, the World will be very soon in a very unsustainable situation with supply of about everything. To assess such scenarios is a major undertaking and would have to wait for a later time with good funding. A better understanding of what is real need that meets the minimum requirement versus “want” or “demand” is needed. The gallium use yield must also be analyzed and assessed whether improvements are possible. A full scenario-analysis exploring the outcome from different policy proposal is needed. The Integrated Assessment Model WORLD7 could be used for developing a multi-goal, multi component policy that is optimized toward sustainability criteria derived from formal definitions and set goals. The Imaginaries are policy proposals that have yet to be embodied with action plans and explored with systems analysis (Causal loop mapping, integrated flow charts, integrated energy charts). Such a process is required and necessary for the Imaginaries to have a foundation in reality.

## Conclusion

The model was used, and there was found a significant risk for technology metal scarcity under business-as-usual scenario. The WORLD7 model was used to simulate the extraction rates, ore grade decline, and approximate metal price levels for gallium. We need to carefully distinguish between the primary production from mines and total supply to the market. The rate of recycling is far too low, and the supply situation may be significantly improved if the recycling rates can be increased substantially. The introduction of extraction methods like heap leaching and subsequent electrowinning for many of the source metals (copper, zinc, lead) yields less of the technology metals and is a real threat to the long-term supply of these metals. These methods are preferred for low and ultralow ores grades and have become more frequent in mining. Market mechanisms have not been able to have sufficient effect, and it appears that governmental interventions are unavoidable. Our simulations demonstrate that gallium scarcity is not immediate, but is likely to develop after 2030 if extraction and recycling yields remain low. Our analysis suggests that pressing for more mining to reduce the demand–supply gap will make short-term improvements at the cost of severe shortage later. A combination of improving extraction yields and improving recycling yields combined with demand reduction may improve the supply situation and push the supply peak beyond 2100. In relation to the EEA imaginaries, each EEA imaginary offers a distinct approach to mitigating gallium scarcity, ranging from conservation-focused strategies to technologically driven solutions. These imaginaries highlight the necessity of tailored policy interventions to achieve the desired future state and avoid critical shortages. They emphasize the importance of understanding and addressing the unique challenges posed by gallium shortages. it is as important to improve yields as it is to find more gallium in deposits. It is as important to improve extraction and recycling yields as it is to find more gallium in deposits.

## Data Availability

No datasets were generated or analyzed during the current study.

## Declarations

### Who did What?

Harald Ulrik Sverdrup developed the gallium module for the WORLD7 model. Harald Sverdrup and Anna Hulda Olafsdottir developed the WORLD7 model as it stands today. Harald Sverdrup wrote up the manuscript draft and did the simulation runs. Harald Sverdrup and Hördur Haraldsson cooperated on finalizing the manuscript, doing revisions and corrections. Hördur Haraldsson worked on the policy implications and future assessments.
